# Patients with secondary amenorrhea due to tuberculosis endometritis towards the induced anti-tuberculosis drug category 1

**DOI:** 10.11604/pamj.2016.24.121.9709

**Published:** 2016-06-07

**Authors:** Raditya Perdhana, Sutrisno Sutrisno, Yani Jane Sugiri, Siti Candra Windu Baktiyani, Arsana Wiyasa

**Affiliations:** 1 Obstetrics and Gynecology Department, Medical Faculty, University of Brawijaya, Malang, Indonesia; 2 Saiful Anwar Public Hospital, Malang; 3 Pulmonology Department, Medical Faculty, University of Brawijaya, Malang, Indonesia

**Keywords:** Tuberculosis, secondary amenorrhea, anti-tuberculosis drug

## Abstract

Tuberculosis (TB) is a disease which can affect various organs, including human's genital organs such as the endometrium. Tuberculosis endometritis can cause clinical symptoms of secondary amenorrhea and infertility. Infertility in genital TB caused by the involvement of the endometrium. The case presentation is 33-year-old woman from dr. Saiful Anwar Public Hospital to consult that she has not menstruated since 5 years ago (28 years old). The diagnosis was done by performing a clinical examination until the diagnosis of secondary amenorrhea due to tuberculosis endometritis is obtained. A treatment by using category I of anti-tuberculosis drugs was done for 6 months, afterward an Anatomical Pathology observation found no signs of the tuberculosis symptoms. Based on that, patient, who was diagnosed to have secondary amenorrhea due to tuberculosis endometritis, has no signs of tuberculosis process after being treated by using category I of anti-tuberculosis drugs for 6 months.

## Introduction

Tuberculosis (TB) is a contagious disease that has become a problem in the world and also the major cause of mortality in developing countries [1]. This disease can affect various organs including human's genital organs. One of the genital TB types is tuberculosis endometrial. Genital TB can occur with a variety of gynecological symptoms such as infertility, menstrual disorders, and chronic pelvic pain. Cases of tuberculosis endometritis can cause clinical symptoms such as secondary amenorrhea and infertility [2]. Secondary amenorrhea is the absence of menstruation for 6 months that occurs in women who previously had regular menstruation [3]. Infertility in genital TB is caused by the involvement of the endometrium [4]. The key to genital TB treatment is a combination adequate drugs dose and an accurate duration. Anti-Tuberculosis Drug Therapy was conducted for over 6 months in patients with genital TB infection that accompanied by infertility. Examination and evaluation performed by laparoscopy or hysteroscopy [5]. In this case, patients with secondary amenorrhea due to tuberculosis endometritis underwent hysteroscopy and anatomical pathology examination to determine the therapeutic efficacy and evaluation of TB infection.

## Patient and observation

**Figure 1:**
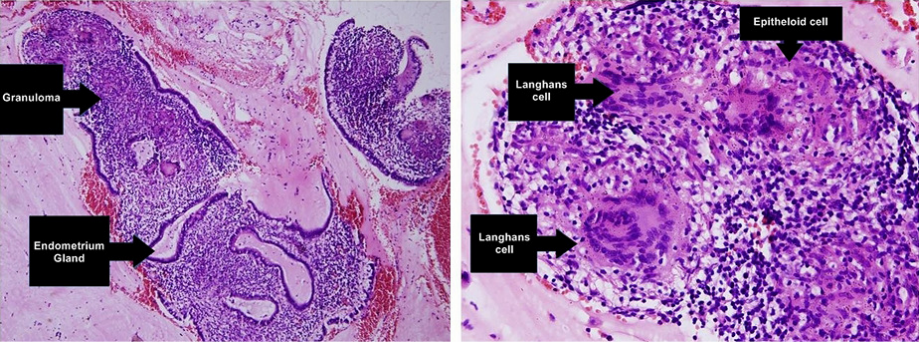
The endometrium prior to administration of anti-tuberculosis drug category I. A) granuloma in endometrial columnar epithelium; B) picture of Langhans cells

**Figure 2:**
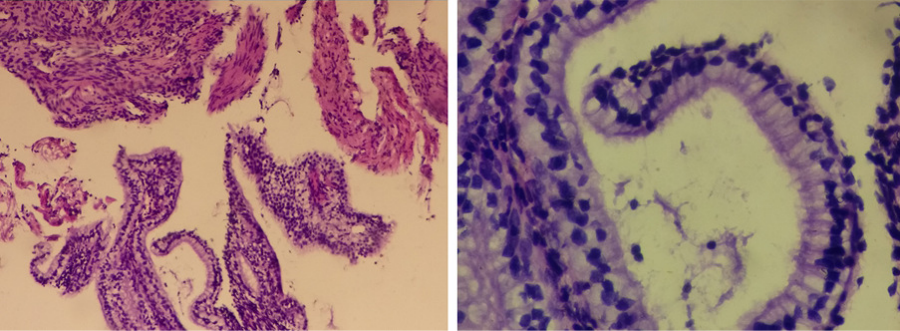
Examination picture of post anti-tuberculosis drug after 6 months

A 33-year-old woman came to Department of Fertility, Endocrinology and Reproduction at Saiful Anwar public Hospital and consulted that she has not menstruated since 5 years ago (28 years old). Her initial menarche was at the age of 14 years and it is in accordance with the normal growth of a child at her age. The patient had a regular menstrual period of 5-7 days, she replaced the pads for 2-3 times / day, and had no menstrual pain. The patient has a history of injective contraceptive that was done every once a month in 2010, and after the injection, she menstruated for two months and then it stopped. Based on clinical examination and a physical examination, the patient was diagnosed with secondary amenorrhea. Investigations that were used are ultrasonography, thorax X-ray and CT Scan Head. Ultrasonography and thorax X-Ray showed that patient is suspected to have right pleural effusion. CT-Scan examination showed that bilateral ethmoidal sinusitis and pituitary gland was within normal limits. The treatment plan in this patient is P test therapy with Prothyra 1x10 mg for 7 days, with control on 2 weeks after the use. P test showed a negative result, because after 14 days of progesterone consumption the prothyra is not bleeding. The treatment is followed by a E+P test (Estrogen+Progesterone) with Estrogen 1x0.625 mg for 21 days and an addition of Progesterone 1x10 mg on 12^th^-21^st^ days, while the control was performed one week after the drug runs out. Based on the E+P test, the obtained results were FSH: 8.71 MIU/mL, LH: 3.1 IU/L, Prolactin: 319.4 ng/mL. The E+P test showed a negative result, so it was necessary to do hysteroscopy and curettage for the uterus evaluation. Hysteroscopy results showed that there were grade 4 adhesions in the uterine cavum, with a pale colored connective tissue in the uterine cavum, which refers to the diagnosis of secondary amenorrhea suspect tuberculosis endometritis, while the result of tissue curettage was examined on the Anatomical Pathology department. Results of anatomical pathology hysteroscopy showed endometrial tissue with stromal looks granulomas with lymphocytes, histiocytes, epithelioid and Langhans multinucleated giant cells and no malignancy was found in this preparation, which showed that patient's diagnosis is secondary amenorrhea suspected tuberculosis endometritis (Figure 1). Based on the Anatomical Pathology hysteroscopy result, treatment was done by using anti-tuberculosis drug category I (FDC 1X3 tab) for 6 months. Anatomical pathology result through macroscopic observation showed visible network of approximately 0.3 cm and 0.4 cm in diameter with a grayish-white color, while the microscopic observations showed fibromuscular tissue at high cylindrical epithelium with polyps shaped vacuolar, and inflammatory granulomatic and aplastic cells were not found (Figure 2).

## Discussion

Mycobacterium tuberculosis is the cause of 90-95% genital TB [6]. TB on genital organs most often occurs in the fallopian tube (90-100% of cases) and in the endometrium (50-80% of cases) [2]. Female's genital tuberculosis is the cause of secondary amenorrhea and infertility [7]. The tuberculosis case in this patient is tuberculosis endometritis that caused clinical symptoms of secondary amenorrhea. A woman is said to have secondary amenorrhea if a woman in reproductive age, who had experienced menstruation, suddenly stops menstruating for at least 3 consecutive months. Secondary amenorrhea is caused by many things, where the diagnosis process requires anamnesis, physical examination, and a thorough investigation. Causes of secondary amenorrhea are generally caused by abnormalities in organs that responsible for the occurrence of the menstrual cycle and the release process of the menstrual blood itself [4]. The patient underwent several tests to determine the diagnosis for the symptoms. The initial examination is a P test (progesterone). The purpose of this test is to assess the level of endogenous estrogen and the genital tract competence. Response to the administration of progesterone was assessed after 2-14 days then the LH serum level was measured [4]. The result of the P test is negative because after the 10^th^ day of drug administration there was no bleeding. P test is said to be positive when there is bleeding on the 2nd-7th day of post administration. Estrogen test was conducted to actively stimulate the development of endometrial and uterine bleeding [4]. This test is performed by administering estrogen for 21 days and the addition of progesterone on the 12^th^-21^st^ day. E+P test was used to examine this patient because the P test is negative. The E+P test is positive when bleeding occurs between 2-3 days after the administration of estrogen and progesterone. In every woman, the use of progesterone and estrogen stimulates different duration and different quantity of bleeding. E+P test negative if there is no bleeding and there is no sign of menstruation. P test and E+P test showed negative results, so the level of LH, FSH, and serum prolactin was measured to determine the function of hormone secretion. The examination showed that FSH and LH levels were normal and there was an increase in serum prolactin levels. Serum prolactin level in these cases showed an increase in serum prolactin levels. Serum prolactin increases due to the hormonal imbalance in the phase of the menstrual cycle that causes menstrual irregularities.

The patient was given the anti-tuberculosis drug category I, after the results of P test and E+P test declared negative. Anti-tuberculosis drug category I that was administered to this patient consists of 2HRZE/4H3R3 (2 months of Isoniazid Rifampicin Piramizid Etambutol/4 months of Isoniazid Rifampicin that was given every three weeks) for 6 months, in which 2 months as the intensive phase and 4 months as the advanced phase. The medication was given every day and continuously monitored in the intensive phase. Evaluation of the provision of anti-tuberculosis drug category 1 was done by hysteroscopy and anatomical pathology examination. The purpose of the hysteroscopy examination was to get a picture of the great adhesion in the uterine cavity [8]. The anatomical pathology examination can be seen from endometrial scrapings material that indicates the presence of Langhans cells and tubercle [9]. Signs of endometrial tissue that was infected with tuberculosis initially were not visible macroscopically. But the caseosa picture and ulceration can be seen later when the development of TB reach an advanced stage. The evaluation by using hysteroscopy examination and anatomical pathology examination showed that infection with TB bacteria was not found after the administration of anti-tuberculosis drug category I. This shows that anti-tuberculosis drug category 1 can provide a clinical improvement in patients with secondary amenorrhea. The administration time of anti-tuberculosis drug therapy determines the success of the therapy. The earlier the infection is found and treated, the better prognosis of clinical improvement in the patient.

## Conclusion

Based on the clinical diagnosis and examination that has been conducted in patient with secondary amenorrhea due to tuberculosis endometritis, it can be concluded that clinical improvement is obtained after administration of anti-tuberculosis drug category 1 for 6 months. This result is proved by the absence of TB infection in the endometrial tissue on the anatomical pathology examination.
